# Quantification of *eef1a1l1* transcript in early zebrafish development

**DOI:** 10.17912/micropub.biology.001944

**Published:** 2026-01-13

**Authors:** Gloria D. Ligunas, Stefan C. Materna

**Affiliations:** 1 Quantitative and Systems Biology Graduate Group, University of California, Merced, Merced, California, United States; 2 Molecular and Cell Biology, University of California, Merced, Merced, California, United States; 3 Health Sciences Research Institute, University of California, Merced, Merced, California, United States; 4 Center for Cellular and Biomolecular Machines, University of California, Merced, Merced, California, United States

## Abstract

*Eukaryotic elongation factor 1-alpha* is among the most highly expressed genes in vertebrates. Because its transcript levels are less sensitive to perturbations than those of other housekeeping genes, the zebrafish ortholog *eef1a1l1* is widely used as a reference. Here, we report absolute measurements of *eef1a1l1* transcript abundance spanning the first 36 hours of zebrafish development. We find that *eef1a1l1* transcripts accumulate rapidly; however, when normalized to cell number, its expression is remarkably steady. This distinguishes *eef1a1l1* from developmentally regulated genes and underscores its suitability as an invariant internal control.

**Figure 1. Quantification of transcript abundance during early zebrafish development:**
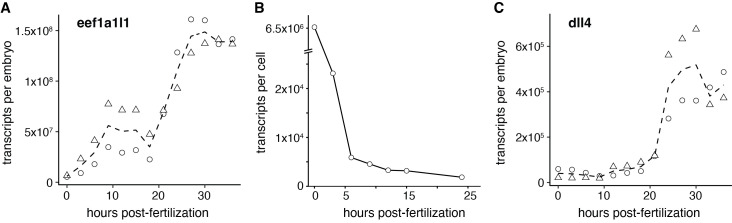
A) Temporal expression profile of *eef1a1l1* at 3-hour intervals. Absolute transcript numbers were measured in two independent clutches (triangles, circles). Dashed line represents mean. B) Abundance of *eef1a1l1* per-cell using mean values shown in (A). After the onset of zygotic expression, *eef1a1l1* levels remain nearly constant when normalized to cell number. C) Temporal expression profile of *dll4* . Zygotic expression commences post-gastrulation but increases considerably between 21 and 24 hpf. Levels of *dll4* per embryo are more than two orders of magnitude lower compared to *eef1a1l1* .&nbsp;

## Description

Housekeeping genes are commonly used as internal references in gene expression studies because their expression is broadly stable across tissues and conditions. Due to their ubiquitous and invariant expression, housekeeping genes are also well suited to be internal standards for estimating transcript levels. While most gene expression measurements are relative, absolute numbers provide a direct measure of gene activity and offer deeper insight into biological relevance. Although absolute transcript numbers can be inferred from spike-in controls in RNA-sequencing experiments (Mortazavi et al., 2008), obtaining such data requires additional effort when using other quantification methods.

The zebrafish *eef1a1l1* gene *(* ZDB-GENE-990415-52), formerly *ef1a* (Gao et al., 1997), encodes a core component of the translation machinery — one of the most abundant proteins in eukaryotic cells (Slobin, 1980; Thiele et al., 1985). In zebrafish, *eef1a1l1* expression is less responsive to perturbations than that of other housekeeping genes (e.g., *gapdh* , *α-actin* ), making it an excellent internal standard in this system (McCurley and Callard, 2008; Tang et al., 2007). Despite its routine use, its own expression has not been studied in detail.

We here determined absolute *eef1a1l1* transcript abundance throughout the first 36 hours of embryonic development. We raised embryos from two independent crosses to the desired time, and, after ensuring proper staging (Kimmel et al., 1995), collected 30 embryos of each batch. To determine absolute numbers of *eef1a1l1 transcripts* , we added precise amounts of two external standards — 10 ^7^ transcripts each of *in vitro* -transcribed *GFP* and *mCherry* RNA — to each lysate, which enabled us to account for differences in RNA extraction and reverse transcription. RNA was then extracted with thorough digestion of genomic DNA, followed by cDNA synthesis and quantitative PCR (qPCR) to determine *eef1a1l1* levels relative to our external standards. We calculated the average C _t_ for *eef1a1l1* and a combined average C _t_ for the two external standards; we determined the relative abundance of *eef1a1l1* to external standards using the ∆C _t_ method&nbsp;(Livak and Schmittgen, 2001), and converted these values into absolute transcript numbers per embryo.

We found that a comparatively large amount of *eef1a1l1* transcript, about 6.5 × 10⁶ copies per embryo, was maternally deposited ( Fig. 1A ). Following the onset of zygotic transcription, abundance of *eef1a1l1* increased through cleavage and gastrulation, then remained constant through mid-somitogenesis (18 hpf), averaging around 7 x 10⁷ transcripts per embryo. Although absolute levels varied between batches, this plateau was consistent across samples. During late somitogenesis, *eef1a1l1* abundance rose sharply: between 18 and 27 hpf, transcript levels doubled to reach approximately 1.37 × 10⁸ copies per embryo, after which they remained stable for the remainder of the sampling period.

We expected *eef1a1l1* abundance to increase steadily throughout early zebrafish development, but its rise was discontinuous. Transcript counts per embryo, however, do not account for differences in cell number. To estimate transcript levels per cell, we normalized *eef1a1l1* abundance using published cell counts from whole-embryo imaging studies. Because no single dataset spans our entire sampling period, we combined values from multiple sources (Keller et al., 2008; Kobitski et al., 2015) and supplemented the 24 hpf time point with an additional estimate (Wagner et al., 2018), which were in close agreement. Beyond this stage, more accurate cell counts would be needed for reliable estimates. Notably, these data indicate that cell numbers plateau after gastrulation, similar to our *eef1a1l1* measurements. In consequence, following the rapid rise in cell numbers during cleavage, *eef1a1l1* levels per cell remain nearly constant until at least 24 hpf ( Fig. 1B ). Overall, *eef1a1l1* transcript abundance per cell behaves as expected of a housekeeping gene — essentially steady.

To compare *eef1a1l1* abundance with that of a developmentally induced gene, we quantified levels of *dll4* transcript. *dll4* (ZDB-GENE-041014-73) encodes one of five zebrafish Delta-like ligands; its earliest expression during embryogenesis is restricted to vascular endothelium, where it promotes arterial differentiation (McCracken et al., 2023; Sacilotto et al., 2013). We first detected zygotic *dll4* expression at 12 hpf, just after endothelial progenitors are first specified ( Fig. 1C ) (Hogan and Schulte-Merker, 2017). At this stage, we estimated ~40,000 transcripts per embryo, a level that remained steady for several hours. By 21 hpf, *dll4* transcript abundance increased sharply to an average of more than 400,000 transcripts per embryo—an approximately ten-fold rise. In contrast, during the same period *eef1a1l1* levels increased only two-fold. This upregulation coincides with the onset of blood flow, which promotes *dll4* expression and arterial differentiation (McCracken et al., 2023). Assuming that the fraction of arterial endothelial cells remains constant — approximately one percent of the embryo (Gurung et al., 2022; Lawson et al., 2001) — this corresponds to a substantial five-fold increase in *dll4* levels per-cell.

The transcript numbers for *eef1a1l1* should be viewed as approximate. Although measurements were consistent across time points, transcript levels differed by as much as two-fold between batches for both *eef1a1l1* and *dll4* , suggesting that biological variation may be considerable. In addition, reverse-transcription efficiencies may differ between in vitro–transcribed standards and endogenous transcripts. However, such differences do not affect the temporal pattern of the expression profile.

Our data reveal that *eef1a1l1* levels per embryo change over time, and that this change is not constant. Small differences in staging will thus introduce more variability during periods of rapid change. Although the need for accurate staging is obvious, small differences are often difficult to avoid. For example, invasive methods such as microinjection often cause notable delays in development that must be accurately assessed. While this is straightforward early in development, this is more challenging post somitogenesis when overt changes of the embryo are more subtle.

In sum, our data illustrate why *eef1a1l1* is an excellent internal control in gene expression analyses. Its levels are remarkably steady on a per cell basis making it insensitive to changes in amount of material sampled. However, *eef1a1l1* levels change over time and the rate of change varies. This underscores the importance of accurate staging when using *eef1a1l1* levels as an internal control.&nbsp;

## Methods

**Animals and Embryos**

Experiments were performed in accordance with an approved IACUC protocol (UC Merced, protocol #2023-1144). For crosses, fish were set up overnight; after pulling the dividers, embryos were collected within 15 minutes to minimize variability. Embryos were grown under standard laboratory conditions (28.5˚C) until sampling at the relevant time point. We collected 30 embryos at each time point and lysed these in 350 µl RLT Plus buffer (Qiagen, Germany). Lysate was stored at –80°C.

**RNA Extraction**

We used *in vitro* transcribed *gfp* and *mCherry* RNA as external standards. These were transcribed from linearized plasmid, quantified, and diluted to a concentration of 10 ^6^ transcripts/µl. After defrosting, right before RNA extraction, we added 10 µl of each RNA solution (10 ^7^ transcripts each), to individual samples. To ensure complete lysis, samples were run through Qiashredder columns before RNA extraction. RNA extraction was performed using the Qiagen RNeasy micro kit including the on-column DNAse digestion step with the following modifications: for on-column DNA removal, we extended incubation to 1 hour at room temperature. We eluted RNA using 100 µl of preheated nuclease free water followed by a 10 min incubation at 60˚C prior to centrifugation.

&nbsp;

**Reverse Transcription**

We reverse transcribed 1 µg of total RNA using Quantabio qScript cDNA synthesis kit according to manufacturer's instructions. Following reverse transcription, we adjusted the sample volume to 200 µl.

&nbsp;

**Quantification**

We determined transcript prevalence by qPCR using Quantabio PerfeCTa SYBR Green FastMix on a Jena Analytik Q-Tower instrument. Per qPCR reaction we used 1 µl of cDNA as prepared above; all reactions were set up in triplicate. We calculated ∆C _t_ values of *eef1a1l1* and *dll4* relative to the combined mean C _t_ of *gfp* and *mCherry* (primer efficiency was between 1.95 and 2 for all primers; primer specificity was assessed by melt-curve analysis immediately following qPCR amplification). ∆C _t_ values were converted to transcript numbers using the 10 ^7^ molecules added as external standard and normalized to the number of embryos sampled to arrive at copies per embryo. Finally, a running average was calculated over three time points. The primers used for qPCR were as follows:

*eef1a1l1* , F: CACGGTGACAACATGCTGGAG, R: CAAGAAGAGTAGTACCGCTAGCAT; primers span the exon 4–5 junction (NM_131263.1)

*dll4* , F: AGTGTGACAGCAGCCCACGC, R: TGGGGAATCTGCGCAGGTGAG; primers span the exon 7–8 junction (NM_001079835.1)

*GFP* , F: GATGGAAGCGTTCAACTAGCA, R: GCAGATTGTGTGGACAGGTAAT

*mCherry* , F: GACCACCTACAAGGCCAAGA, R: CTCGTTGTGGGAGGTGATGT

We used the following time–cell number pairs (hpf: cells): 0:1, 3:1,000, 6:7,000, 9:17,000, 12:21,700, 15:22,500, and 24:50,000. Values for 0–15 hpf are based on Kobitski et al. (2015) and are consistent with Keller et al. (2008); the 24 hpf estimate was reported by Wagner et al. (2018).
